# Comparison of complications and surgery outcomes in skin closure methods following cesarean sections

**DOI:** 10.1007/s00404-024-07911-6

**Published:** 2025-01-25

**Authors:** Daniel Gabbai, Chen Jacoby, Itamar Gilboa, Sharon Maslovitz, Yariv Yogev, Emmanuel Attali

**Affiliations:** Department of Obstetrics and Gynecology, Lis Hospital for Women’s Health, Tel Aviv Sourasky Medical Center, Tel Aviv University, Tel Aviv, Israel

**Keywords:** Cesarean delivery, Closure methods, Surgical outcomes

## Abstract

**Purpose:**

This study aimed to assess the impact of absorbable subcutaneous staples for skin closure in cesarean delivery (CD) on maternal morbidity.

**Methods:**

A retrospective cohort study was conducted at a single tertiary university-affiliated medical center between January 2011 and April 2022. In 2020, a new technique involving absorbable subcutaneous staples for skin closure in CD was introduced. We compared surgical outcomes among three groups: non-absorbable staples, absorbable subcutaneous staples, and absorbable subcutaneous sutures. Data were compared between the three groups and demographic, obstetric, and surgical characteristics were analyzed using univariate and multivariate analysis.

**Results:**

During the study period, 31,660 CDs were performed in our center. The data of 31,419 CDs were available for analysis. Absorbable subcutaneous staples were associated with a significantly shorter surgery time in comparison to non-absorbable staples and sutures (52 min vs 53 min vs 60 min, *p* < 0.001). No differences were found in rates of wound infections or any surgical site surgery in the 45 days following CD. In a multivariate analysis: the use of absorbable subcutaneous staples was associated with a significantly lower risk for prolonged hospitalization > 5 days (OR 0.6, *p* < 0.001) and re-admission within 45 days (OR 0.8, *p* = 0.04).

**Conclusion:**

The use of absorbable subcutaneous staples for skin closure during CD is associated with shorter surgery times and a lower risk of prolonged hospitalization and readmission within 45 days.

## What does this study add to the clinical work

**Table:** 

This study demonstrates that absorbable subcutaneous staples as a skin closure method for cesarean delivery significantly reduce surgery time, hospitalization duration, and readmission rates compared to traditional methods, offering a safe and efficient alternative for optimizing surgical outcomes.	

## Introduction

Cesarean delivery (CD) is one of the most common surgeries worldwide, with a prevalence of one in three women in the United States [1]. According to the American College of Obstetricians and Gynecologists (ACOG), CD significantly increases a woman’s risk of pregnancy-related morbidity and mortality [2].

Complications related to cesarean skin wounds impact 3–15% of surgeries, encompassing issues such as superficial infection, wound separation, and the accumulation of fluids like seroma or hematoma. These complications necessitate extended scar care, and in certain instances, they may result in prolonged maternal hospitalization and the need for surgical intervention [3].

Metallic staples, subcuticular sutures, and biological glue are all accepted methods for skin closure in CD (4). An increased risk of wound complications and prolonged hospitalization following skin closure with absorbable sutures compared to staples was reported [4]. On the other hand, other reports [5, 6] suggested that closure of skin with subcutaneous suture decreases the composite wound complication by 50% compared with staples. Besides surgery time, it has also been suggested that different skin closure techniques, do not influence postoperative or long-term cosmetic outcomes [7]. Moreover, pain at 6 weeks postoperatively was significantly less following skin closure with staples compared to subcuticular suture [8].

The utilization of absorbable subcuticular staples, a relatively new skin closure technique, is a convenient, safe, and cost-effective method [9]. However, randomized controlled trials (RCTs) failed to reveal clinically significant differences between this method and traditional skin closure techniques [10]. Yet, previous reports included a relatively small number of patients and failed to report long-term complications of different techniques for skin closure.

Thus, we aimed to compare surgical outcomes associated with skin closure using staples, sutures, and absorbable staples within a large-scale population.

## Material and methods

### Subjects and study design

We conducted a retrospective cohort study of all women who underwent CD in a single, University-affiliated tertiary medical center, between January 2011–April 2022.

Those who had missing data regarding the skin closure method were excluded. All deliveries were categorized into three groups according to the closure method: (a) non-absorbable staples; (b) absorbable subcutaneous staples; (c) absorbable subcutaneous sutures.

The choice of skin closure method was based on the surgeon’s preference and medical contraindications.

A comparison was made between the demographic and clinical characteristics as well as indications for CD between the three groups.

The study was approved by the Institutional Review Board (IRB TLV-0284-08, July 28th, 2021).

### Cesarean delivery protocol

In our center, the standard protocol for abdominal closure is as follows: The uterus is meticulously closed using a double-layer technique with Polyglactin The closure of the abdominal fascia involves continuous suturing using either Polyglactin or Polydiozanone Suture (PDS), while the subcutaneous layer is closed discontinuously only if it measures 2 cm or more depth.

The choice of skin closure, whether through subcuticular sutures, staples, or absorbable staples, is determined based on the surgeon's preference.

### Data collection

All medical charts were reviewed from the computerized delivery room logbooks. The database encompassed demographic, obstetric, and clinical characteristics such as maternal age, parity, gravidity, previous CD, gestational age at delivery, conceiving method, multiple pregnancy, and gestational diabetes in the index delivery. Moreover, CD features such as surgical techniques and medical indications for CD were included. Surgical outcomes such as prolonged hospitalization (defined as 5 days of postoperative hospitalization or more), re-admission, re-operation, and wound complications were retrieved.

Surgery time was calculated from the patient's entry into the operating room until the end of the surgery. Preterm delivery was determined as delivery before 37 weeks of gestation. Grand-multiparity was defined as the history of five or more births (live or stillborn).

Instances involving re-admission, re-operation, and wound complications were obtained.

The study was approved by the Institutional review board (IRB TLV-0284-08, July 28th 2021).

### Statistical analysis

Univariate analysis techniques were used to identify differences between the groups. The One-way Anova and Kruskal Wallis (in the case of non-normally distributed variables) were used to assess the significance of continuous variables. In the same way, we used the Chi-square test (or Fisher’s exact test, as needed) for categorical variables. A multivariable logistic regression model, controlling variables that were found to be statistically different between groups, was used to determine the role of the independent variables.

The data were analyzed using Statistical Package for the Social Sciences (SPSS) software (version 21.0; SPSS Inc., Chicago, IL, USA). A *p*-value < 0.05 was considered significant. Data analysis was performed with an unidentified database. Therefore, informed consent was not needed.

## Results

During the study period, a total of 31,660 cesarean deliveries were performed in our center, with 31,419 of these deliveries being available for comprehensive analysis. Among these, non-absorbable staples were employed in 22,932 instances (71.6%), absorbable subcutaneous staples in 7305 instances (23.9%), and absorbable subcutaneous sutures in 1384 cases (4.5%).

Demographic, obstetrical, and surgical characteristics are presented in Table 1. Notably, women within the non-absorbable staples group exhibited a higher BMI in comparison to the absorbable subcutaneous staples and absorbable subcutaneous sutures (ASSu) groups [23.2 (20–26.9) vs. 22.6 (20.4–22.8) and 21.7 (19.7–24.6), respectively, *p* < 0.001). Furthermore, cases in the absorbable subcutaneous sutures cohort demonstrated a reduced prevalence of severe preeclampsia and gestational diabetes in comparison to the other groups (3.3% vs. 4.3–5.4% and 12.4% vs. 17.2–19.3%, *p* < 0.001, respectively).

**Table 1:** Demographic, obstetric, and labor characteristics of the studied population

	NAS *N* = 21,932	ASSt *N* = 7305	ASSu *N* = 1384	*p*-value
Maternal age (mean, SD)	34.43 (5.2)	34.67 (5.2)	34.9 (4.7)	**< 0.001**
BMI (median, IQR)	23.2 (20, 26.9)	22.6 (20.4, 22.8)	21.7 (19.7, 24.6)	**< 0.001**
GDM *n* (%)	3770 (17.2)	1411 (19.3)	171 (12.4)	**< 0.001**
Severe PET *n* (%)	1186 (5.4)	313 (4.3)	46 (3.3)	**< 0.001**
Grand multiparity *n* (%)	275 (1.3)	65 (0.9)	15 (1.1)	**0.034**
ART pregnancies (%)	4792 (23.2)	1453 (22.9)	287 (23.4)	**0.87**
Multiple gestation *n* (%)	3487 (15.9)	795 (10.9)	223 (16.1)	** < 0.001**
Preterm Labor *n* (%)	4017 (18.3)	839 (12.2)	217 (15.7)	** < 0.001**
Urgent CD *n* (%)	10,791 (50.2)	3595 (50.1)	628 (46)	**0.023**
CD duration, (median, IQR)	53 (44, 64)	52 (43, 63)	60 (50, 71)	** < 0.001**
Hypodermis closure *n* (%)	6015 (31.4)	5079 (70.1)	1133 (85.3)	**< 0.001**
Bladder Flap *n* (%)	19,844 (90.5)	6733 (92)	1272 (91.9)	**< 0.001**
Sharp peritoneal dissection *n* (%)	3117 (14.2)	659 (9)	154 (11.1)	**< 0.001**
General anesthesia *n* (%)	1684 (7.7)	215 (2.9)	38 (2.7)	**< 0.001**
Length of stay > 5 days, n(%)	1986 (8.6)	333 (4.6)	92 (6.6)	**< 0.001**
Wound infection, *n* (%)	84 (0.4)	35 (0.5)	6 (0.4)	**0.54**
Any surgery within 45 days *n* (%)	137 (0.6)	35 (0.5)	8 (0.6)	**0.36**
CD-related surgery (including re-laparotomy), *n* (%)	42 (0.2)	9 (0.1)	0 (0)	**0.49**
Wound-related surgery, *n* (%)	565 (2.6)	143 (2)	30 (2.2)	**0.01**
Re-admission (any reason), *n* (%)	555 (2.5)	139 (1.9)	30 (2.2)	**0.007**

*NAS* Non-Absorbable Staples, *ASSt* Absorbable Subcutaneous Staples, *ASSu* Absorbable Subcutaneous Sutures. *SD* Standard Deviation, *IQR* Interquartile range, *N* Number, *GDM* Gestational Diabetes Mellitus, *CD* Cesarean Delivery, *NRFHR* non-reassuring fetal heart rate, *PET* Pre-eclampsia

The longest duration of CD was observed in the ASSu group, with a median of 60 (IQR 50–71) minutes, followed by the NAS group at 53 min (IQR 44–64), and the ASSt group at 52 min (IQR 43–63) (*p* < 0.001).

After applying a multivariate analysis (Table 2), controlling for multiparity, BMI, gestational diabetes, pre-eclampsia, multiple pregnancies, preterm birth, surgery time, urgent CD, anesthesia type, and surgery characteristics, the utilization of absorbable subcutaneous staples emerged as a protective factor for readmissions within 45 days post CD (OR = 0.8, 95% CI 0.6–0.9), as well as prolonged hospitalization (OR = 0.6, 95% CI 0.5–0.68).

**Table 2:** Multivariate analysis after adjustment for multiparity, BMI, gestational diabetes, pre-eclampsia, multiple pregnancies, preterm birth, surgery time, urgent CS, anesthesia type and surgery characteristics

	Adjusted OR	*p*-value
Re-admission (any reason) *n* (%)		
NAS	1	
ASSt	0.8 (0.6–0.9)	**0.04**
ASSu	0.84 (0.5–1.3)	**0.43**
Hospitalization > 5 days *n* (%)		
NAS	1	
ASSt	0.6 (0.5–0.68)	**< 0.001**
ASSu	0.8 (0.6–1.07)	**0.145**

*OR* Odds Ratio, *NAS* non-absorbable staples, *ASSt* absorbable subcutaneous staples, *ASSu* absorbable subcutaneous sutures.

Skin closure technique did not exhibit any associations with the prevalence of wound infections or any CD-related surgeries within 45 days following delivery (Table 1).

## Discussion

In this study, our objective was to examine surgical outcomes in association with various skin closure methods. The key findings of our research are (1) ASSt closure correlated with a reduced duration of CD; (2) Notably, ASSt closure is an independent protective factor for prolonged hospitalization and readmission within 45 days after CD.

### Our findings in the context of other observations

Traditionally, skin closure in cesarean sections has been achieved through NAS and ASSu. In the last decade, ASSt has been introduced and incorporated into clinical practice [11]. Consequently, measuring the global prevalence of ASSt usage presents a challenge. Within our cohort, ASSt has emerged as the predominant technique for skin closure in CD, accounting for an overall prevalence of approximately 24%.

In our study, we identified ASSt use as a protective factor for hospitalization exceeding 5 days following the surgery. To our knowledge, this specific outcome has not been thoroughly investigated in the context of skin closure methods. The observed association may be elucidated by various factors; for instance, previous studies have established a correlation between wound complications and prolonged hospitalization after CD [12] highlighting concerns related to healthcare system issues [13].

Wound complications play a cardinal role in the postoperative care of women undergoing CD. Several studies [4, 5] sought to identify the optimal skin closure technique to reduce the prevalence of complications. While wound complications were found to be in lower incidence with subcuticular staple closure versus traditional staple closure [9], Madsen et al. demonstrated no differences in wound complications when using absorbable subcuticular staples compared to subcuticular sutures [10].

An alternative explanation could be rooted in the necessity for post-operative pain control following CD [14]. While ASSu and NAS were found to be comparable in terms of analgesic usage after CD [15], skin closure with ASSt was associated with decreased analgesic use compared to NAS [11].

Another finding in our study is the reduced risk of readmission following CD when utilizing ASSt. The literature has highlighted various risk factors for this complication, including infections [12], notably those related to wound complications, and the length of stay following CD [16]. Both identified risk factors offer possible explanations for the association we observed.

In addition to surgical complications, the duration of surgery carries both medical and economic implications [17]. Various studies have endeavored to compare the duration of CD procedures, focusing on common techniques. Cochrane's investigation into maternal and operative outcomes revealed a shorter surgery time when using NAS compares to ASSu [18], a finding supported by a meta-analysis conducted by Tulli et al. [19]. Conversely, Madsen et al. did not identify a significant difference in surgery time between ASSu and ASSt [10].

In contrast, our study demonstrated a shorter duration among the groups, particularly within the two absorbable techniques. This variance can be attributed to the experience and preferences of the surgeons involved. Given the substantial number and heterogeneity of surgeons in our study, we believe that our results possess external validity.

### Clinical implications

CD has become one of the most prevalent surgeries, making the selection of optimal surgical approaches vital for enhancing patient outcomes. Moreover, given the frequent occurrence of CD, reducing surgery time could lead to improved scheduling and accessibility at healthcare facilities. These aspects hold both medical and economic importance. Thus, from our vantage point, adopting this skin closure method is of great clinical significance for CD practices.

### Strengths and limitations

Our study exhibits several strengths. First, it addresses a crucial clinical inquiry concerning the method that has evolved as the predominant skin closure technique in CD. While preceding research, as previously referenced, our study distinguishes itself by possessing the largest sample size to evaluate these outcomes. Secondly, by being a single-center medical study, we were able to meticulously compare a homogenous cohort of CDs, enhancing the internal validity of our findings. Third, univariable analysis was performed to assess candidate variables as risk factors for prolonged hospitalization and re-admission, and multivariate logistic regression confirmed the independent associations of ASSt utilization with these specific outcomes.

Being a retrospective cohort study, it is imperative to acknowledge several inherent limitations. Notably, our capacity to control all potential confounding factors was restricted, such as the surgeon’s proficiency and expertise. Furthermore, the acquisition of data from computerized chart records rendered us susceptible to information bias, a consideration that might influence the integrity of our findings.

## Conclusions

This study revealed that utilizing absorbable subcutaneous staples for skin closure during CD is associated with shorter surgery durations and a decreased likelihood of prolonged hospital stays and readmissions within 45 days. We believe that adopting this technique could bring about both medical and economic benefits.

## Data Availability

No datasets were generated or analysed during the current study.
